# Multiple Approaches of Neuro-Physiotherapy Used for Improving Balance, Normalizing Tone, and Gait Training in a Child With Ataxic Cerebral Palsy: A Case Report

**DOI:** 10.7759/cureus.50264

**Published:** 2023-12-10

**Authors:** Dhanashree S Upganlawar, Snehal Samal, Prishita Koul, Jaee P Kapre

**Affiliations:** 1 Neuro Physiotherapy, Ravi Nair Physiotherapy College, Datta Meghe Institute of Higher Education and Research, Wardha, IND

**Keywords:** cerebral palsy, case report, rood’s approach, ataxic cp, pelvic pnf, cimt, physiotherapy

## Abstract

Cerebral palsy (CP) is a non-progressive developmental delay disorder that mainly affects children. A strategy for enhancing organizational abilities, including practices based on evidence, and improving outcomes is the base of clinical management in physiotherapy. A seven-year-old girl presented with a history of difficulty walking, standing for extended periods, and performing gross and fine motor movements. MRI revealed generalized atrophy of the cerebellum. The child was managed by medications and physiotherapy. Physiotherapy intervention was based on goal-oriented strategies, which include Rood’s approach, constraint-induced movement therapy (CIMT), proprioceptive neuromuscular facilitation (PNF), passive stretching, etc. This goal-oriented program showed an improvement in the treatment outcomes of the child. The child was walking independently with a proper gait pattern and was able to maintain both static and dynamic balance. Initial physical therapy management using integrated methods promotes the achievement of developmental milestones like gross motor skills in ataxic cerebral palsy children.

## Introduction

The developmental impairment disorder is known as cerebral palsy (CP). It is non-progressive and takes place in children. William Little originally gave it a description in the 1840s. The term CP is used as a generalized umbrella term for a variety of neurological diseases that affect posture and mobility due to early-stage brain injury [1]. There are two to three and a half cases of CP per 1000 births worldwide. CP presents a variety of clinical presentations, aetiologies, severity levels, comorbidities, and outcomes, making it a complex and multifaceted condition [2]. Around 5-10% of children have ataxic CP, which is a neurological condition. CP is much more common in preterm neonates [3].

Children affected with cerebellar ataxic CP typically have underdeveloped or abnormalities of the cerebellum, which makes it difficult for the cerebellum to integrate the neuronal data needed to control movement and balance smoothly [4]. Cerebellar ataxic CP children show unusual movement performance with non-typical force, rhythm, and accuracy, poor coordination, fine motor issues, dysmetria, generalized hypotonia, and intention tremors, and have poor postural control because of a disproportion between their agonist and antagonist muscles, a weak equilibrium, and defensive reactions [5,6]. Due to weakened abdominal and back muscles, CP children have poor core stability and there is a link between functional activity and core stability [7].

For children with CP, a variety of therapy approaches are available. Physiotherapy techniques include range of motion, stretching, strengthening, etc; these all are traditional techniques for treatment. A paradigm for enhancing organizational capability, including best practices based on evidence, and improving outcomes is clinical management in physiotherapy [8]. To enhance balance and posture, mobilization of the trunk and pelvis as well as active trunk extension is also encouraged by the therapist. Apart from these effective treatments, hydrotherapy, which includes alternating movement of the lower limbs, and therapeutic riding on a horse are also suggested [8].

## Case presentation

A full-term seven-year-old female child presented who was born from vaginal delivery normally without any complications after birth. When the child was six months old, she suffered from pyrexia and breathlessness, and for that, she visited a local hospital. She was diagnosed with pneumonia and managed conservatively for the same. She had a recurrent history of pneumonia until she turned five years of age. At three years of age, she had difficulty standing independently. After her milestones started to delay (walking, gross and fine motor movements) for all, the patient’s mother took the patient to a private hospital where she was treated conservatively. For further management, the patient was brought to a tertiary care hospital.

Clinical findings

The child was ectomorphic in build. The child was cooperative, conscious, and oriented. On observation, both the hips were internally rotated, the knees slightly flexed, and the ankle was slightly in plantar flexion. On postural examination, the patient was made to stand. In the anterior view, both anterior superior iliac spines (ASISs) were not on the same level, and hip hiking on the right side was seen. In lateral view, the pelvis was anteriorly tilted, and there was increased lumbar lordosis and genu recurvatum of both knees. In the posterior view, neither posterior superior iliac spine (PSIS) was on the same level.

Clinical diagnosis

MRI of the brain was carried out and suggested that there was generalized atrophy of the cerebellum. The study also revealed that there was a mild change in the flocculonodular lobe. According to clinical features, it was a suggestive case of ataxic cerebral palsy.

Physiotherapy functional assessment

On examination, the tone was hypotonic for both the lower limbs and normal for both upper limbs. The range of motion for the upper limb was normal and for the lower limb, it was reduced. Manual muscle testing (MMT) grading for the upper extremity was good (Grade 4), i.e., near normal as compared to the lower extremity (Grade 3) according to the Medical Research Council (MRC). Deep tendon reflexes were normal for the upper limb and diminished for the lower limb. In both lower extremities, there was a tightness of the Piriformis, adductors, Hamstring muscles, and Achilles tendon. When the child was made to walk, the gait was ataxic.

Outcome measures were taken before initiating the treatment as shown in Table 1. Primary outcome measures were gross motor function classification system (GMFCS) and manual muscle testing (MMT). Secondary outcome measures including functional independence measures for children (WeeFIM), pediatric balance scale (PBS), and manual ability classification system (MACS) were used to evaluate the patient.

**Table 1:** Outcome measures taken before initiating the treatment GMFCS: Gross Motor Function Classification Scale, MMT: Manual Muscle Testing, WeeFIM: Functional Independence Measure for Children, PBS: Pediatric Balance Scale, MACS: Manual Ability Classification System

Primary outcome measures	Scores (day 1)
GMFCS	Level 2
Manual Muscle Testing (MMT)	Hip extensors - 3/5; Knee extensors -3/5; Plantar flexors - 3/5
Secondary outcome measures	Scores
WeeFIM	95/126
PBS	37/56
MACS	Level 2

Physiotherapy intervention

**Figure 1:**
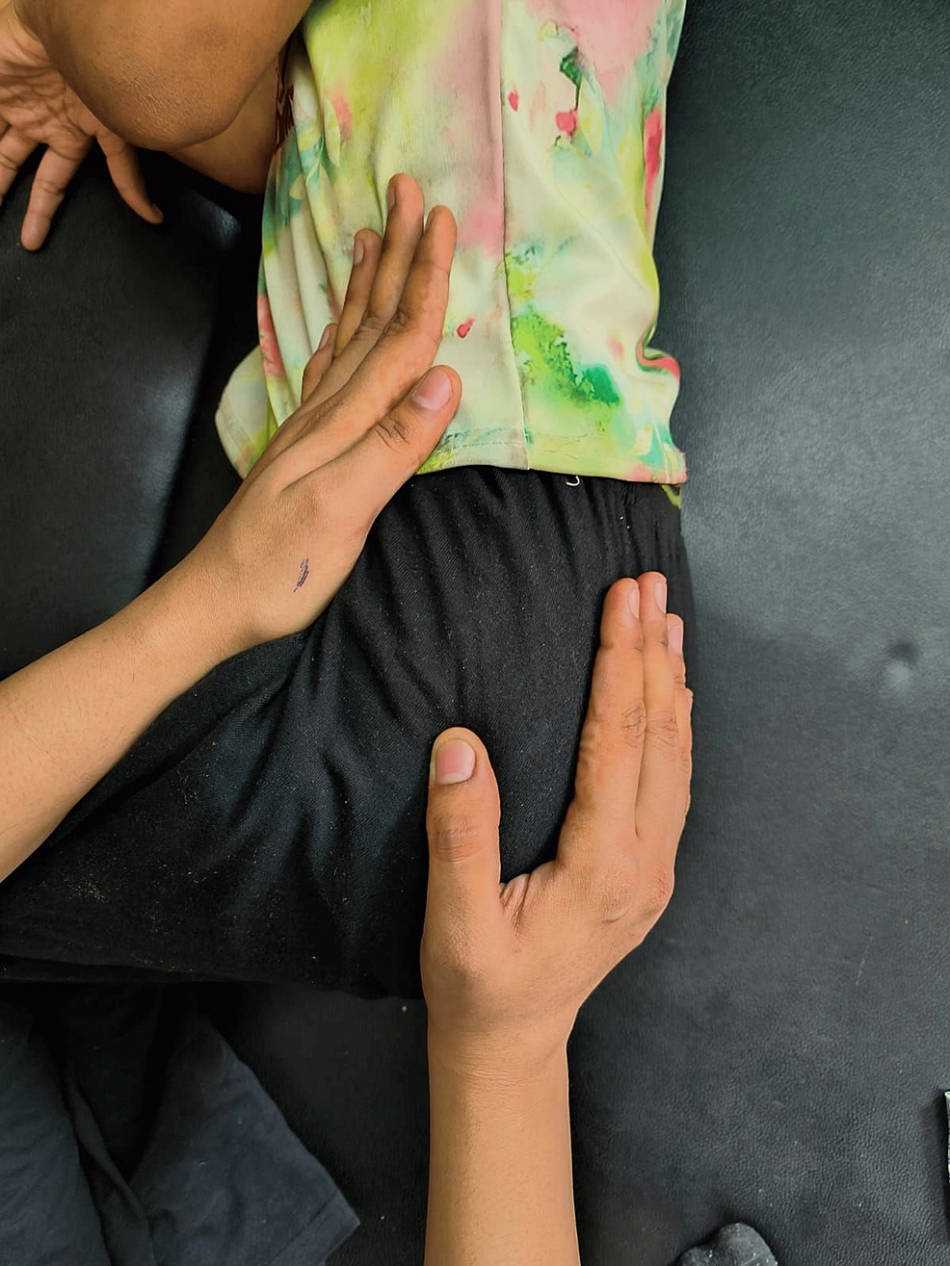
Posterior approach of pelvic PNF PNF: Proprioceptive neuromuscular facilitation

**Figure 2:**
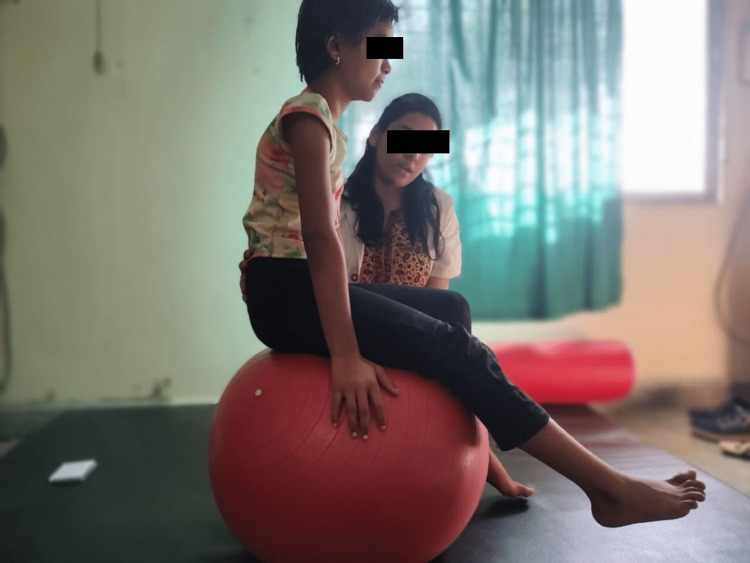
Dynamic quads on vestibular ball

**Figure 3:**
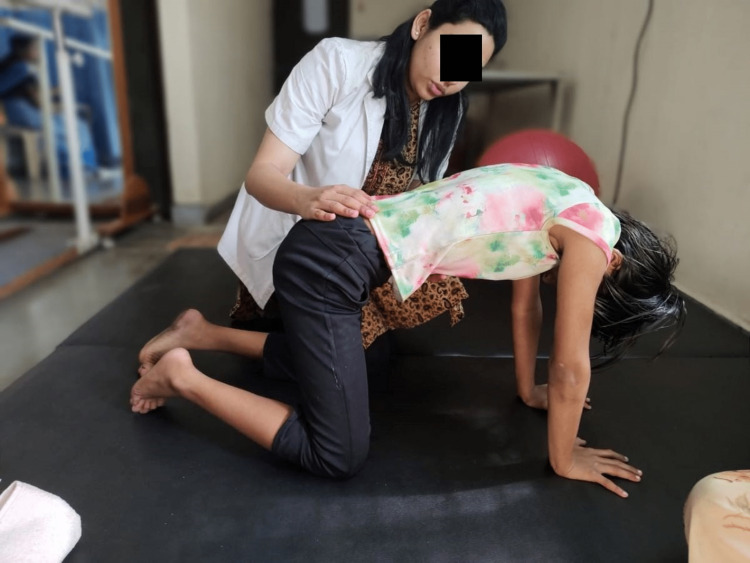
Camel exercise for posture correction

**Figure 4:**
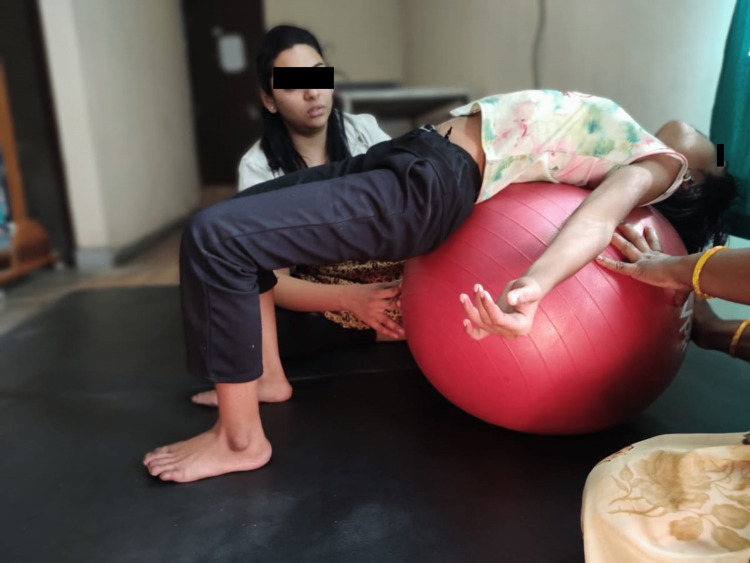
Pelvic bridging on vestibular ball

**Figure 5:**
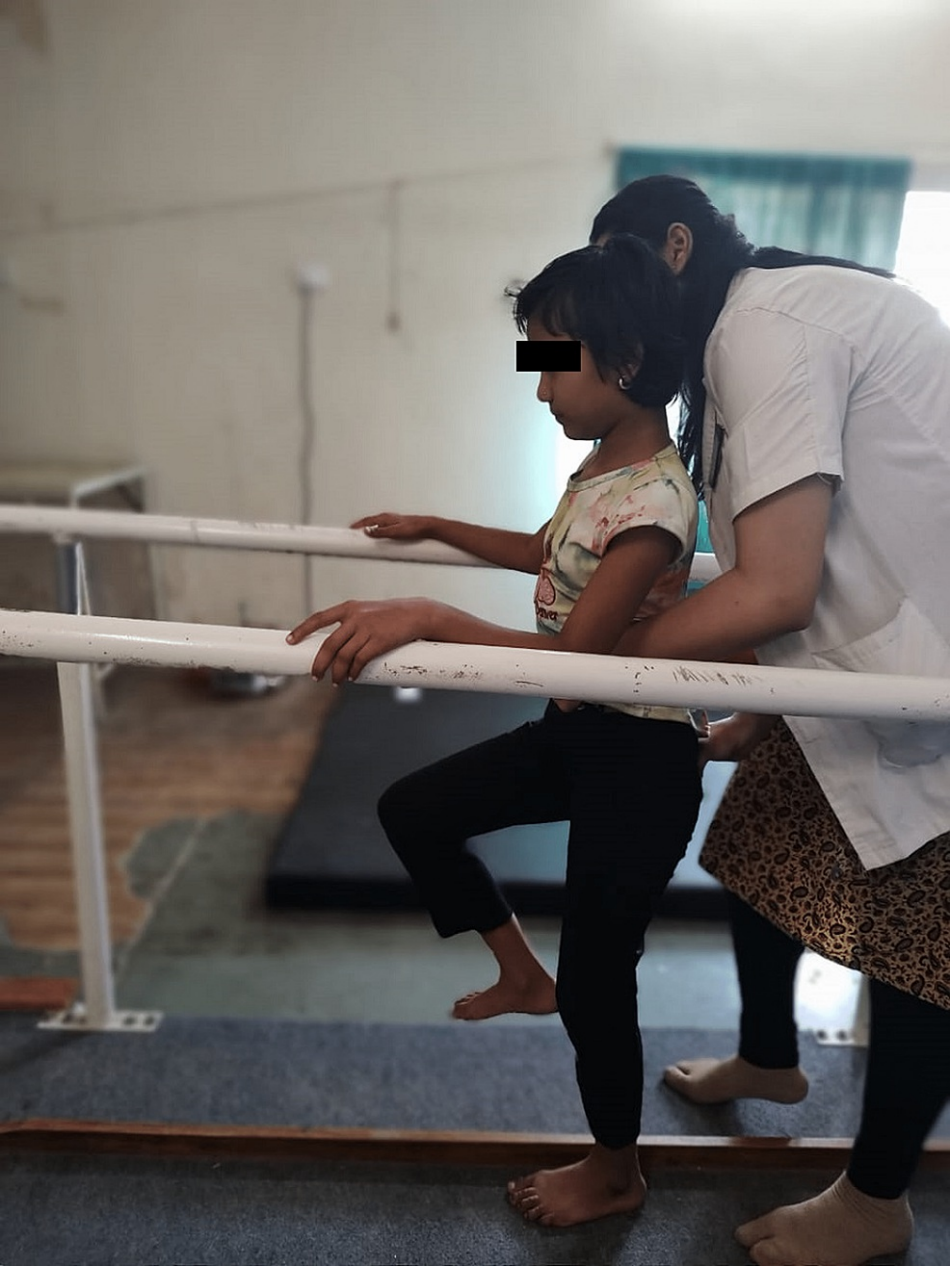
Spot marching for gait training

Physiotherapy management was focused on improving muscle tone, restoring normal pelvic congruence, strengthening of muscles, improving balance, etc. Various strategies were used for the above-mentioned goals, some of them are Rood's approach, constraint-induced movement therapy (CIMT), proprioceptive neuromuscular facilitation (PNF), CP standing frame, etc. Table 2 indicates the physiotherapy rehabilitation protocol (Figures 1-5).

**Table 2:** Sequence of physiotherapy protocol LL: Lower limbs, PNF: Proprioceptive neuromuscular facilitation, AT: Achilles tendon, secs: seconds, mins: minutes, VMO: Vastus medialis obliquus, CP: Cerebral palsy, CIMT: Constraint-induced movement therapy

Problem identified	Goal	Strategies	Intervention
Hypotonia in LL	To improve the reduced muscle tone (hypotonia of LL)	Rood’s approach [9]	A quick stretch and heavy joint compression were used to facilitate the tone of the lower limbs. 20 repetitions of each approach for 15 mins.
Pelvic malalignment	Restoring normal pelvic congruence	Pelvic PNF	The posterior depression technique of pelvic PNF using slow reversals as it is anteriorly tilted. 5-sec hold was given with 20 repetitions for 10 mins.
Tightness of hamstrings, adductors, AT, and piriformis muscles	To reduce the tightness	Sustained stretching	Passive sustained stretching of the hamstring, adductors, TA, and piriformis was given. 30-sec hold with 3 repetitions for 5 mins.
Weakness of quadriceps and abductor muscles	To strengthen the muscles	Strengthening protocol	Dynamic quads on vestibular ball. 5-sec hold with 20 repetitions. VMO strengthening, Abductor muscles strengthening. 5-sec hold with 20 repetitions for 10 mins.
Lordotic posture	To improve posture	Vestibular ball rehabilitation	Camel exercises were given to the patient. 5-sec hold with 20 repetitions for 5 mins. Pelvic bridging on the vestibular ball (Figure 3). 10-sec hold with 20 repetitions for 5 mins (Figure 4).
The patient was unable to stand for a prolonged time	To maintain and improve standing balance	CP standing frame	The patient was made to stand by stabilizing the patient with a belt around the CP standing frame for 2 minutes and progression was made to 4 min, 6 min, and so on.
Balance was affected	To improve the balance	CIMT [10]	CIMT technique was used to train the balance of the child. The child was asked to stand on one leg, reach out activities with one leg, etc.
Ataxic gait	To improve the gait	Gait training using pelvic PNF	Gait training was initiated in parallel bars in front of a mirror so that the child could get feedback. Pelvic PNF was also used to train the gait parameters. Squatting was taught. spot marching was started to train gait. Stepping one step forward and backward. Spot marching; all these were taught (Figure 5).

Follow-up and outcomes

After six weeks of an integrated neuro-physiotherapy approach which included CIMT, PNF techniques, stretching, strengthening, and Rood's approach, the child gained good outcomes. At the end of four weeks, there was enhanced tone and normal tone was achieved. There was an enhancement in other outcome measures as well such as improvement in GMFCS from level 2 to level 1. Manual muscle testing grade was also improved from 2/5 to 4/5 at the end of six weeks. Child balance and gait also improved a lot (Table 3).

**Table 3:** Post-treatment outcome measure score GMFCS: Gross Motor Function Classification System, MMT: Manual Muscle Testing, PBS: Pediatric Balance Scale, WeeFIM: Functional Independence Measure for children, MACS: Manual Ability Classification System

Primary outcome measures	Week 2	Week 4	Week 6
GMFCS [11]	Level 2	Level 1	Level 1
MMT	Hip extensors- 3+/5; Knee extensors-3+/5; Plantar flexors -3+/5	Hip extensors- 4/5; Knee extensors-4/5; Plantar flexors- 4/5	Hip extensors- 4+/5; Knee extensors- 4+/5; Plantar flexors- 4+/5
Secondary outcome measures	Week 2	Week 4	Week 6
PBS [11]	42/56	49/56	54/56
WeeFIM	100/126	110/126	120/126
MACS	Level 2	Level 1	Level 1

## Discussion

In this instance, physiotherapy management was created using integrated approaches, such as static weight-bearing exercises, passive stretching, goal-oriented approaches, Rood's approach, CIMT, etc. It was then assessed using the Modified Ashworth Scale (MAS), PBS, GMFCS, MACS, and WeeFIM [8].

The therapy had a positive effect on the child. Her remarkable session compliance was likely a result of the treatment strategy and strategies adopted, which included engaging in entertaining activities throughout the sessions. CIMT was found to be very effective in improving the balance of the patient in the Tedal et al. study. Our treatment approach was also in line with this study [12].

The favorable outcomes of the treatment protocol also included the parents as they carried out some of the basic exercises at home, and the patient was consistent with the treatment. The treatment carried out in the home was walking, standing on one leg, climbing one stair up and down, and squatting [13]. For improving gait parameters and pelvic alignment, the pelvic PNF technique was used regularly for better outcomes, and in the study by Shah et al., it was shown to be effective and the same result was obtained in our study [14]. To normalize the tone, quick stretch, and heavy joint compression were used as facilitatory techniques and were proven effective in normalizing tone as the Patel et al. study suggested. We used the same technique and the results were effective in our case too [15].

Systematic reviews have shown that physical treatment regimens based on balance training and muscular strengthening in cerebellar ataxia patients enhance balance and lessen the severity of ataxia. In our research, we used a strengthening procedure, and the findings were consistent with the field of study [16]. According to this analysis, specific muscle groups with some gains in GMFCS and function provide the greatest evidence for the effectiveness of strengthening programs. In this case study, the vastus medialis oblique, quadriceps, adductor muscles, and other muscles were strengthened. The outcome was consistent with the direction of the study [17].

In this case study, the child was given a task-specific program that included tasks like standing on one leg, reaching out, walking forward, walking sideways, etc. This program increased the patient's GMFCS score. Our findings corresponded with those of Salem and Godwin's study [18]. Following a six-week rehabilitation program, the caregiver was given instructions on how to perform exercises with repetitions and holds at home. The caregiver received education regarding the value of home protocol. This was in accordance with the research done by Bailes et al. [19].

## Conclusions

This case report comes to the conclusion that children with ataxic CP benefit from the integration of early neuro-physiotherapy with a target-oriented treatment plan such as passive stretching, static weight-bearing exercises, CIMT, pelvic PNF, Rood's approach, and task-oriented approaches. Both clinically and in terms of the outcome metrics, the child improved. After six weeks, the child was able to perform gross and fine motor movements, walk with near to normal gait patterns, maintain balance, and decrease lordosis. After continuing the treatment at home two months later, the child was able to walk independently with proper gait patterns.
